# Pre-Emptive OCT-Guided Angioplasty of Vulnerable Intermediate Coronary Lesions: Results from the Prematurely Halted PECTUS-Trial

**DOI:** 10.1155/2020/8821525

**Published:** 2020-12-03

**Authors:** Jan-Quinten Mol, Michiel J. Bom, Peter Damman, Paul Knaapen, Niels van Royen

**Affiliations:** ^1^Department of Cardiology, Radboud University Medical Center, Nijmegen, Netherlands; ^2^Department of Cardiology, Amsterdam UMC, Vrije Universiteit Amsterdam, Amsterdam, Netherlands

## Abstract

**Objectives:**

To assess the safety and efficacy of pre-emptive treatment of optical coherence tomography- (OCT-) derived vulnerable, non-flow-limiting, non-culprit lesions in patients with myocardial infarction (MI).

**Background:**

Intracoronary imaging with OCT can aid in the decision to treat non-flow-limiting lesions by identifying vulnerable plaques. Pre-emptive treatment of these lesions might improve patient outcome by “sealing” these plaques. Bioresorbable vascular scaffolds (BVS) have theoretical benefit for this treatment because they dissolve completely over time.

**Methods:**

In patients presenting with MI, non-culprit lesions with a fractional flow reserve ≥0.8 were imaged with OCT. Vulnerable plaques were randomised to either percutaneous coronary intervention (PCI) with bioresorbable vascular scaffold (BVS) placement or optimal medicinal therapy (OMT). The primary outcome was a composite of all-cause mortality, non-fatal MI, and unplanned revascularisation at 1-year follow-up.

**Results:**

The trial was stopped prematurely after retraction from the market of the Absorb BVS. At that time, a total of 34 patients were randomised. At two years, the composite endpoint occurred 3 times (18.8%) in the BVS group and 1 time (6.3%) in the OMT group. Apart from one elective PCI for stable angina in the OMT group, no target lesions in any group were revascularised.

**Conclusions:**

Pre-emptive stenting of vulnerable plaques had no evident benefit compared to conservative treatment. However, due to the low number of included patients, no definite conclusions could be drawn. Identifying and potentially treating vulnerable plaques remains an important target for future research. This trial is registered under https://www.trialregister.nl/trial/NL4177 on 08-12-2015.

## 1. Introduction

Although percutaneous coronary intervention and pharmacologic therapies have improved the prognosis for patients with acute coronary syndrome (ACS), recurrent major adverse cardiac events (MACE) are still common. Such events may occur at the original culprit lesion site, at pre-existing non-culprit lesions or at newly developed lesions. For non-culprit lesions, intracoronary physiology can guide in the decision to perform stent placement. However, the MACE rate in ACS patients after FFR-guided deferral of stent placement in these non-culprit lesions is high as compared to stable coronary artery disease (CAD) [1].

It has been advocated that anatomic plaque characterization using intracoronary imaging could further aid the treatment decision for non-culprit lesions in ACS. Several pathology studies have shown that there are certain morphological plaque characteristics that make an atherosclerotic plaque vulnerable to rupture and subsequent myocardial infarction (MI). These lesions generally have a large necrotic core and a thin overlying fibrous cap and are frequently referred to as thin-cap fibroatheromas (TCFA) [2]. These features have also been prospectively identified in vivo. In the PROSPECT study, a cohort of ACS patients underwent three-vessel intravascular ultrasound (IVUS) for the assessment of plaque morphology during the index invasive coronary angiography [3]. After a median follow-up of 3.4 years, it was shown that non-culprit lesions identified as TCFA had a hazard ratio of 3.35 for causing future MACE. Nevertheless, the use of IVUS for assessing plaque components is hampered by its relatively limited spatial resolution. In pathology studies, the average fibrous cap thickness of ruptured plaques at autopsy was 23 *μ*m, and 95% of all ruptured caps measured less than 65 *μ*m [4], whereas IVUS only has a spatial resolution of 100 *μ*m. Since PROSPECT, Optical Coherence Tomography (OCT) intracoronary imaging has become available. OCT has a much higher spatial resolution of approximately 10 *μ*m, which truly allows for measurement of fibrous cap thickness. Additionally, OCT is able to identify infiltrating macrophages, which is another hallmark of unstable lesions [5]. This intravascular imaging technique, therefore, seems to be more suitable for the evaluation of plaque morphology [6].

Identifying vulnerable plaques in vivo has led to the hypothesis of preventive intervention of these lesions. A small study has shown that stenting TCFA resulted in the development of neointima, thereby “sealing” the vulnerable plaque [7]. A bioresorbable vascular scaffold (BVS) might have a theoretical benefit for this treatment because it dissolves completely over time. Therefore, we initiated the PECTUS trial, a multicenter randomised controlled trial comparing PCI with BVS (Absorb) placement versus Optimal Medicinal Therapy (OMT) of OCT-determined vulnerable, FFR-negative, non-culprit lesions in patients with myocardial infarction (NL4177).

The trial was stopped prematurely after the reporting of increased stent-thrombosis and the subsequent retraction from the market of the Absorb stent [8, 9]. At that time, a total of 34 out of the 500 planned patients were enrolled in the trial. Here, we report the OCT characteristics and the clinical outcomes of these 34 patients.

## 2. Materials and Methods

### 2.1. Study Flow

**Figure 1:**
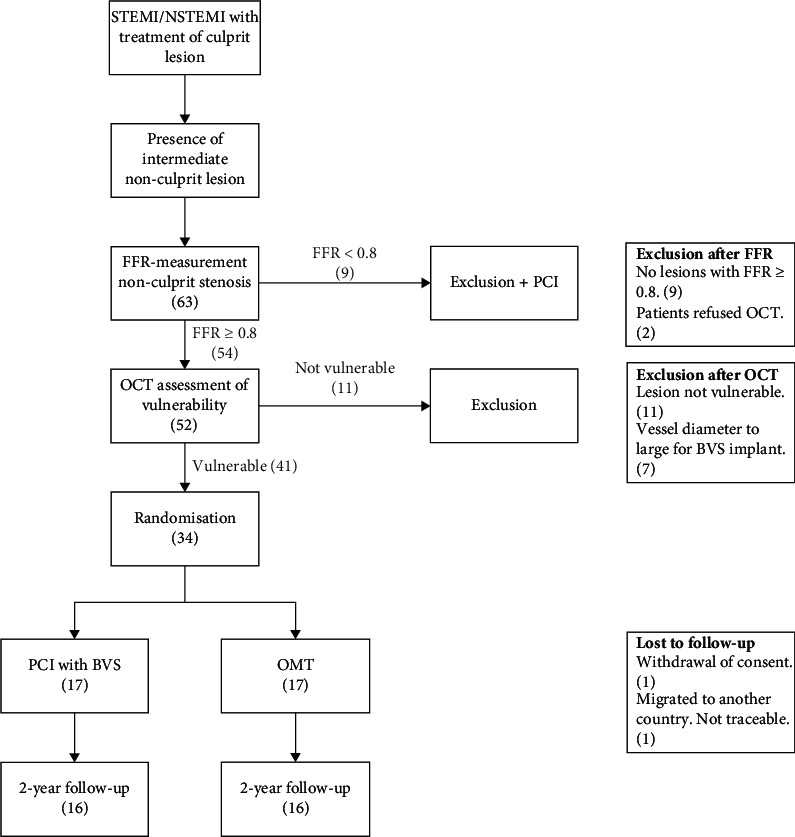
Study Flowchart. BVS, bioresorbable vascular scaffold, FFR, fractional flow reserve, NSTEMI, non-ST-elevation myocardial infarction, OCT, optical coherence tomography, OMT, optimal medicinal therapy, PCI, percutaneous coronary intervention, STEMI, ST-elevation myocardial infarction.

A flowchart of the study design is shown in Figure 1. Patients presenting to the hospital with a myocardial infarction (ST-elevation or a non-ST-elevation) were screened for potential inclusion in the study. Patients were treated according to the current guidelines for the management of ACS, including referral for invasive coronary angiography (ICA) and (potential) PCI of the culprit artery. Patients were subsequently approached for participation in the study if any intermediate non-culprit stenoses were identified during ICA and if they were eligible for inclusion based on the criteria listed in Table 1.

**Figure 2:**
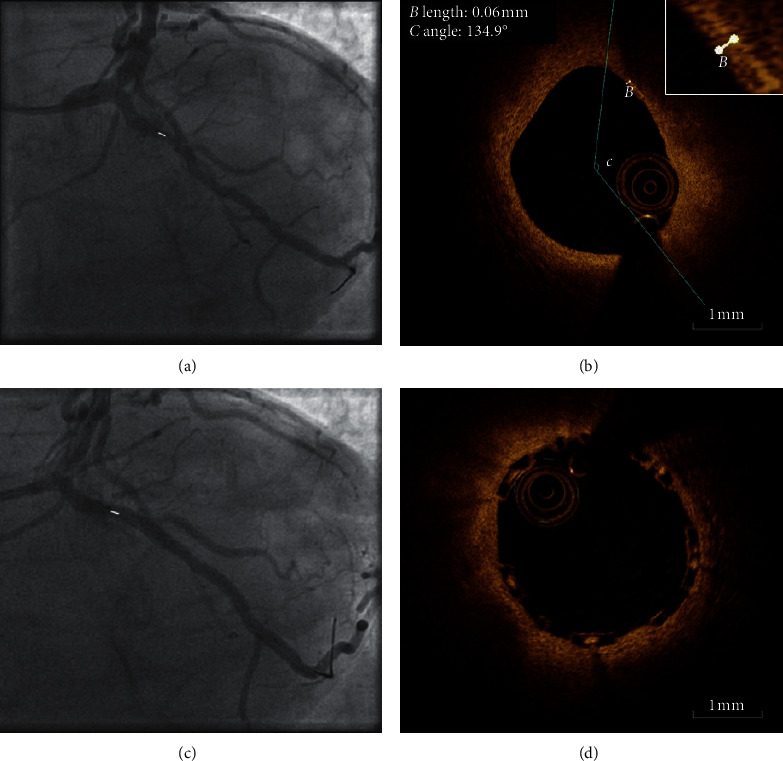
OCT with angiography coregistration of a patient randomised to BVS placement. (a) Angiogram shows an intermediate stenosis in the proximal circumflex coronary artery (white marker). (b) OCT of the stenosis reveals a vulnerable plaque (lipid arc >90° with a cap of 60 *μ*m). (c) Angiogram shows the same lesions as in A (white marker) after BVS placement. (d). OCT shows BVS placement over vulnerable plaque with good stent apposition.

After obtaining informed consent, patients underwent a re-ICA during which the non-culprit lesions were evaluated for physiological significance by fractional flow reserve (FFR). FFR-positive (<0.80) lesions were treated with PCI according to current guidelines. All non-flow-limiting (FFR ≥ 0.80) lesions were imaged with OCT. OCT Images were acquired using a commercially available frequency-domain OCT system (Abbott, formerly St Jude Medical, USA). Trained personnel evaluated the OCT images of target lesions for plaque vulnerability. Vulnerable plaques were randomised to either PCI and subsequent stenting with a BVS (Figure 2) or optimal medical therapy in a 1 : 1 fashion. No more than one lesion per patient was randomised, even if multiple vulnerable plaques were identified. The choice which lesion to target was left at the discretion of the operator, but preferably, the most proximal lesion was selected and with consideration of the following hierarchy: first, the left anterior descending artery and, second, the right coronary artery in patients with a right dominant coronary circulation or the left circumflex artery in patients with a left dominant circulation. Patients with only FFR-positive or only non-vulnerable lesions were excluded from the study protocol and subjected to clinical follow-up in a registry.

### 2.2. OCT Analysis

OCT analysis of target lesions was performed in real time during the ICA. Analysis of any additional “non-target” lesions that were captured on the OCT pullback was performed offline using an offline review workstation (Abbott, formerly St Jude Medical, USA). Evaluation of the images was based on tissue characteristics as previously described in OCT expert consensus papers [10]. A plaque was deemed “vulnerable” if it contained two of the following characteristics: a lipid arc (defined as a diffusely bordered signal-poor region) of more than 90 degrees, a cap thickness (defined as a signal-rich band overlying a lipid core) of <65 *μ*m, and either cap rupture or thrombus formation.

### 2.3. Medicinal Therapy

Patients in both groups received pharmacologic treatment according to current guidelines. Due to the reporting of increased stent-thrombosis of BVS compared to metallic stents during the trial, all randomised patients were recommended to continue dual antiplatelet therapy (DAPT) for at least 3 years if they tolerated DAPT without bleeding complications and were not at high bleeding risk (e.g., prior bleeding on DAPT, coagulopathy, or oral anticoagulant use).

### 2.4. Outcome Measure and Power Analysis

The primary outcome of this trial was a composite of all-cause mortality, non-fatal MI, and unplanned revascularisation at 1-year Secondary outcomes included the composite outcome at 2 and 5 years and the individual components at 1, 2, and 5 years. Based on an expected event rate of 5% in the intervention group versus 13% in the OMT group, with a two-sided alpha of 5% and a drop-out rate of 10%, 500 patients were needed to demonstrate superiority of BVS stenting of vulnerable plaques.

## 3. Results

Inclusion in the study started in March of 2016 and was subsequently halted in March of 2017. A total of 63 patients underwent re-ICA with FFR-measurements of non-culprit lesions (Figure 1). In nine patients, the non-culprit lesions were FFR-positive, and hence, subsequent revascularisation was performed. Of the remaining 54 patients, 52 underwent OCT imaging of a total of 59 FFR-negative lesions, whereas 2 patients refused OCT imaging. Among the 52 that underwent OCT imaging, a vulnerable target lesion could be identified in 41 (79%) patients. Of all 59 OCT pullbacks of FFR-negative lesions, 44 (75%) showed a vulnerable lesion. Seven patients were excluded due to a vessel diameter too large for BVS implantation, resulting in a total of 34 patients who were eventually randomised (17 BVS vs. 17 OMT). Apart from one withdrawal of consent in the BVS group and one lost to follow-up in the OMT group, two-year follow-up was obtained of all participants that were included up to that point.

Table 2 shows patient baseline characteristics. The mean age of participants was 62.1 ± 10.4 years in BVS vs. 70.3 ± 5.9 years in the OMT group. The percentage of male participants was 75.0% in BVS vs. 75.0% in OMT. STEMI was the initial presentation in 43% in BVS vs. 50% in OMT. OCT characteristics of the target lesions in the randomised groups (Table 3) were as follows: the average fibrous cap thickness was 50.0 *μ*m ± 10.3 in the BVS group vs. 50.6 *μ*m ± 10.0 in the OMT group. A plaque rupture or thrombus was identified in 1 (6.25%) of the target lesions in the BVS group vs. 2 (12.5%) in the OMT group. 100% of target lesions in both groups contained a lipid arc of >90 degrees. The average minimal lumen area was 2.69 ± 0.99 mm^2^ in the BVS group and 2.57 ± 1.07 mm^2^ in the OMT group. In the BVS group, the average minimal lumen area changed from 2.69 ± 0.99 mm^2^ to 6.55 ± 1.81 mm^2^ after stent placement. Analysis of the OCT images beyond the angiographically targeted lesions revealed the average presence of 0.2 ± 0.4 additional vulnerable non-target lesions and 0.7 ± 1.1 additional non-vulnerable non-target lesions per OCT-pullback in the BVS group. In the OMT group, an additional 0.5 ± 0.8 vulnerable non-target lesions and 0.9 ± 0.7 non-vulnerable non-target lesions were identified.

During two-year follow-up, a total of 12 clinical events were recorded, of which 4 were adjudicated as MACE (Table 4). The number of MACE was 3 in the BVS group and 1 in the OMT group. The events in the BVS group were a non-cardiac death and two non-study vessel-related MIs. The sole MACE in the OMT group was a non-cardiac death. Apart from one elective PCI for stable angina in the OMT group, no target lesions in any group were revascularised during the two-year follow-up.

## 4. Discussion

In the PECTUS trial, patients with myocardial infarction and an FFR-negative OCT-based vulnerable non-culprit lesion were randomised between an intervention with a BVS and optimal medical therapy. Due to the low number of included patients, no robust conclusions can be drawn with respect to the safety or efficacy of preventive stenting of these plaques based on this study. Nevertheless, there were some notable results. In the 34 patients that were randomised, MACE occurred 3 times (18.8%) in the BVS group and once (6.3%) in the OMT group. This would indicate that stenting of vulnerable lesions with BVS has no evident benefit compared to medical treatment or might even result in worse outcomes. However, no case of MACE was related to a target lesion. The only target lesion-related event was an elective PCI due to stable angina in the OMT group. This is in contrast with the AIDA trial [8], in which patients undergoing PCI were randomised between BVS and metallic stents. In this trial, target vessel failure at 2-year follow-up had occurred in 11.7% of patients with BVS. Additionally, no device thrombosis was seen in the current study. This could suggest that BVS implantation is related to relatively low device-related complications in these non-flow-limiting non-complex lesions. This is also in line with the midterm results of BVS in STEMI-patients [11].

We observed a lower-than-expected rate of MACE (6.3%) in the OMT group. In comparison, the 2-year follow-up results of the FAME trial showed a MACE rate of 17.9% in patients who had undergone complete FFR-guided revascularisation [12]. A possible explanation for these discrepancies, in addition to the limited patient population, could be that patients in this trial were advised to continue DAPT for up to 3 years after the index event.

Current guidelines recommend physiological measurements to determine the hemodynamic significance of angiographic intermediate lesions, based on several large trials [13, 14]. Despite the obvious benefit of FFR-measurements in guiding revascularisation, long-term follow-up patients with deferred lesions still show significant event rates. The 5-year follow-up of the FAME trial demonstrated a 28% event rate in patients who had been treated with FFR-guided complete revascularisation [15]. Additionally, some studies have suggested that FFR-guided treatment decision based on studies in patients with stable CAD cannot simply be adopted in ACS patients [1, 16]. Therefore, besides treatment or referral based on physiological significance, anatomical characteristics might further improve treatment decision. In support of this, recent multicentre non-invasive imaging studies have clearly shown that the use of anatomical imaging with coronary computed tomography angiography (CCTA) leads to a more favourable prognosis as compared to standard of care using functional testing [17]. Additionally, a recent small single-center trial demonstrated superiority of image-guided intervention compared with physiology-guided intervention [18]. Although these results have to be confirmed in larger trials, they hint at the additional value of anatomical information in guiding coronary intervention.

In PECTUS, a plaque was considered eligible for randomisation if it showed two characteristics of vulnerability. Whilst all randomised target lesions contained a lipid arc of >90 degrees, only 9% of these lesions showed a plaque rupture or thrombus. Even though these, and other characteristics such as neointimal vascularisation and fibrous cap inflammation, have all been associated with plaque rupture, there is no clear answer as to what (combination of) feature(s) is the most hazardous. Nonetheless, the body of evidence linking plaque morphology to MACE is growing. Following PROSPECT, the VIVA and ATHEROREMO-IVUS studies also showed an association between IVUS-derived characteristics of vulnerability and MACE [3, 19, 20]. Moreover, this association is also found in other imaging modalities such as near infrared spectroscopy (NIRS) and CCTA [21, 22]. Because of the novelty of the technique, validation studies for the ability of OCT to detect vulnerable plaques are scarce. In the recent CLIMA study, investigators analysed images of 1003 patients who had undergone OCT-imaging of the proximal LAD in the context of a clinically indicated ICA. This study showed that the combination of four morphological plaque features was an independent predictor of MACE with a hazard ratio of 7.54 at 1 year [23]. In CLIMA, however, the OCT imaging was performed on a fixed segment of the coronary artery tree as opposed to a segment based on angiographic stenosis. Therefore, the technique was evaluated more as a tool for prognostic risk stratification instead of imaging based lesion treatment. The same holds true for the other intravascular imaging studies. Data on “targeted” intravascular imaging of angiographically defined stenoses is still lacking. Several studies are currently trying to address this gap in knowledge. Among them are the COMBINE study (NCT02989740) and PECTUS-obs study (NCT03857971).

In line with the current PECTUS trial, the abovementioned FORZA trial also assessed OCT-guided PCI [18]. In this study, 350 patients were randomised between FFR-guided or OCT-guided PCI of angiographically intermediate coronary lesions. At 13-month follow-up, patients in the OCT-guided group had significantly less MACE or significant angina compared to patients in the FFR-guided group. However, the decision whether or not to stent a lesion in the OCT group was based on a combination of OCT-derived area stenosis, minimal lumen area, and the presence of plaque rupture. Therefore, apart from plaque rupture, this study did not take characteristics of vulnerability into account. In contrast, two other randomised studies that are currently being conducted have a similar design to PECTUS. The PROSPECT-ABSORB (NCT02171065) and PREVENT (NCT02316886) both randomise patients with residual non-obstructive vulnerable plaques to either BVS placement or optimal medicinal therapy although they differ in the intravascular imaging modality used.

## 5. Limitations

The main limitation of the PECTUS trial is the low number of included patients due to the early termination of the trial.

## 6. Conclusions

In conclusion, the PECTUS trial could not shed light on the theoretical benefit of preventive stenting of non-flow-limiting vulnerable lesions due to early termination of the trial. Nonetheless, the concept of identifying and potentially treating vulnerable plaques remains an important field for future research given the relatively large MACE rates as reported for this specific cohort of patients.

## Figures and Tables

**Table 1:** Inclusion and exclusion criteria.

Inclusion criteria	Exclusion criteria
*Clinical*	*Clinical*
(i) Age ≥18 years	(i) Pregnancy
(ii) Clinical presentation of STEMI or NSTEMI	(ii) Severe kidney disease defined as an eGFR <30 ml/min
	(iii) Previous CABG
	(iv) Indication for revascularization by CABG
	(v) Estimated life expectancy <1 year

*Angiographical*	*Angiographical*
(i) Presence of residual, intermediate CAD (diameter stenosis of 30–90%), with the possibility of plaque vulnerability	(i) Target vessel diameter <2.5 mm or >4.0 mm
	(ii) Anatomy of lesion unsuitable for OCT catheter crossing or imaging (aorta-ostial lesions, small diameter segment, and severe calcifications)
	(iii) Anatomy unsuitable for BVS placement (left main, bifurcation, and side branch (>2 mm) involvement)
	(iv) Target lesion is
	** **(a) In-stent restenosis
	** **(b) Chronic total occlusion
	** **(c) In the same vessel as treated culprit lesion
	** **(d) In the same segment as a previously implanted stent/scaffold

BVS, bioresorbable vascular scaffold, CABG, coronary artery bypass grafting, CAD, coronary artery disease, NSTEMI, Non-ST-elevation myocardial infarction, OCT, optical coherence tomography, STEMI, ST-elevation myocardial infarction.

**Table 2:** Baseline characteristics.

	BVS (*n* = 16)	OMT (*n* = 16)
Age—years	62.1 ± 10.4	70.3 ± 5.9

Sex—*n* (%)		
** **Male	12 (75)	12 (75)
** **Female	4 (25)	4 (25)

Clinical presentation—*n* (%)		
** **STEMI	7 (43)	8 (50)
** **NSTEMI	9 (57)	8 (50)

Target vessel—*n* (%)		
** **LAD	6 (37.5)	7 (43.75)
** **Cx	6 (37.5)	6 (37.5)
** **RCA	4 (25)	3 (18.75)

Average FFR	0.90 ± 0.07	0.90 ± 0.06

BVS, bioresorbable vascular scaffold, Cx, circumflex artery, LAD, left anterior descending artery, FFR, fractional flow reserve, NSTEMI, Non-ST-elevation myocardial infarction, OMT, optimal medicinal therapy, RCA, right coronary artery, STEMI, ST-elevation myocardial infarction,

**Table 3:** Target lesion OCT characteristics.

	BVS (*n* = 16)	OMT (*n* = 16)
Average cap thickness—*μ*m	50.0 ± 10.3	50.6 ± 10.0
Plaque rupture or thrombus—*n* (%)	1 (6.25%)	2 (12.5%)
Lesions with >1 lipid quadrant—*n* (%)	16 (100%)	16 (100%)
Average MLA—mm^2^	2.69 ± 0.99	2.57 ± 1.07
Average MLA after PCI—mm^2^	6.55 ± 1.81	—
Average number of additional non-target lesions per pullback	0.9 ± 1.3	1.4 ± 0.8
(i) Vulnerable	0.2 ± 0.4	0.5 ± 0.8
(ii) Non-vulnerable	0.7 ± 1.1	0.9 ± 0.7

BVS, bioresorbable vascular scaffold, OCT, optical coherence tomography, OMT, optimal medicinal therapy, MLA, minimal lumen area, PCI, percutaneous coronary intervention.

**Table 4:** Clinical events.

No.	Group	Target segment	FFR of target lesion	Time after randomisation	Event	MACE	Target lesion related
1 (1)	BVS	LAD-mid (7)	0.82	During BVS implant	Cardiac arrest due to pulseless electrical activity during BVS implantation, for which chest compressions were performed for 1 minute and atropine was given, after which return of spontaneous circulation occurred. No mechanical complication was seen. Postprocedural troponin values were not elevated. Episode was attributed to a vagal reaction.	No	Yes

1 (2)	BVS	LAD-mid (7)	0.82	6 months	Elective PCI of proximal and distal RCA (in-stent restenosis distal RCA) because of progressive angina. No pre-intervention FFR was performed because stenosis in distal RCA was 90%.	No	No

2	BVS	LAD-mid (7)	0.81	4 months	Cardiac arrest due to ventricular fibrillation. ICA shows left main coronary artery occlusion, for which PCI was performed. OCT shows good patency of BVS in the mid LAD.	Yes	No

3	BVS	LAD-mid (7)	0.84	24 months	Non-cardiac death due to obstruction hydrocephalus caused by metastasized lung carcinoma.	Yes	No

4	BVS	RCA-prox (1)	0.93	23 months	Elective PCI of LAD-mid because of stable angina.	No	No

5	BVS	LAD-mid (7)	0.86	8 days	Infected hematoma of the femoral puncture site/closure device.	No	No

6	BVS	Cx-mid (13)	0.95	15 months	STEMI with PCI of distal RCA (culprit). Additional occlusion of a small MO2 branch. This occlusion was not intervened upon as patient was free of complaints after PCI of the RCA. BVS in the mid-Cx was patent.	Yes	No

7	OMT	LAD-mid (7)	0.87	12 months	Non-cardiac death due to aspiration pneumonia in patient with lymphoma and metastasized squamous cell carcinoma.	Yes	No

8	OMT	Cx-mid (13)	1.00	24 months	Hospital admission with chest pain and slightly elevated cardiac troponin levels without rise/fall. ICA showed no obstructive coronary artery disease. Complaints were attributed to hypertension.	No	No

9	OMT	Cx-prox (1)	0.83	22 months	Lobectomy for newly diagnosed lung carcinoma.	No	No

10	OMT	MO1 (12)	0.97	Same day as randomisation	Transient binocular diplopia after ICA.	No	No

11	OMT	LAD-mid (7)	0.89	24 months	Elective PCI of the mid LAD (target segment) and proximal RCA due to stable angina and optimisation for esophageal cancer-related chemotherapy. No pre-intervention FFR was performed because the wire could not pass the mid-LAD.	No	Yes

BVS, bioresorbable vascular scaffold, Cx, circumflex artery, FFR, fractional flow reserve, ICA, invasive coronary angiography, LAD, left anterior descending artery, MACE, major adverse cardiac event, MLA, minimal lumen area, MO, obtuse marginal artery, OCT, optical coherence tomography, OMT, optimal medicinal therapy, PCI, percutaneous coronary intervention, RCA, right coronary artery, STEMI, ST-elevation myocardial infarction.

## Data Availability

The data used to support the findings of this study are available from the corresponding author upon request.

## Abbreviations

OCT: Optical coherence tomography
MI: Myocardial infarction
PCI: Percutaneous coronary intervention
FFR: Fractional flow reserve
BVS: Bioresorbable vascular scaffold
OMT: Optimal medicinal therapy
ACS: Acute coronary syndrome
MACE: Major adverse cardiac events
CAD: Coronary artery disease
TCFA: Thin-cap fibroatheroma
IVUS: Intravascular ultrasound
STEMI: ST-elevation myocardial infarction
NSTEMI: Non-ST-elevation myocardial infarction
ICA: Invasive coronary angiography
DAPT: Dual antiplatelet therapy
CCTA: Coronary computed tomography angiography
NIRS: Near-infrared spectroscopy
CABG: Coronary artery bypass grafting
MLA: Minimal lumen area.
